# The role of long-term doxycycline in patients of idiopathic pulmonaryfibrosis: The results of an open prospective trial

**DOI:** 10.4103/0970-2113.53231

**Published:** 2009

**Authors:** Parthasarathi Bhattacharyya, Saikat Nag, Sujan Bardhan, Dipabali Acharya, Rantu Paul, Rana Dey, Malabika Ghosh, Ratna Dey, Indranil Saha

**Affiliations:** *Institute of Pulmocare and Research, R. G. Kar Medical College and Hospital, Kolkata, India*; 1 *Department of Community Medicine, R. G. Kar Medical College and Hospital, Kolkata, India*

**Keywords:** Idiopathic pulmonary fibrosis, doxycycline, matrix metalloprotein

## Abstract

**Objective:**

To evaluate the effect of long term use of doxycycline in IPF patients.

**Materials and Methods:**

Patients of IPF, selected randomly from out patient services and diagnosed on the basis of HRCT chest, were put on doxycycline in an open prospective trial. They were followed up with monitoring of subjective well being along with measurement of pulse rate and arterial oxygen saturation at rest and after a fixed and certain exercise, forced vital capacity, six minutes walk test, St Georges Respiratory questionnaire, and serial chest X-rays.

**Results:**

Out of seven patients put on doxycycline, six of them continued the drug for a mean period of 531.43 (± 328.88 days). All the patients tolerated the drug well and had shown uniform subjective and overall objective improvement in all the parameters concerned; the change in the radiological parameter being statistically significant.

**Conclusion:**

Doxycycline merits an appropriate clinical trial in the management of idiopathic pulmonary fibrosis. This widely used and relatively safe drug can add a new dimension to the therapeutic regimen. However, further in-depth studies will be required to evaluate its role in the management of IPF.

## INTRODUCTION

Idiopathic pulmonary fibrosis (IPF) is the clinical counterpart of the usual interstitial pneumonia in the pathological classification of idiopathic interstitial pneumonia.[1] The pathogenesis of IPF is more a kind of dyseregulated fibrogenesis[2] than inflammation induced fibrosis.[3] Since, after diagnosis, the five years survival is barely 40%[4] and there is no good therapy available, any new treatment of IPF is a welcome news. Dysregulated matrix metalloproteinase (MMP) activity following epithelial injury has been found to be related to the up regulation in the release of profibrotic growth factors that are important for continuous stimulation of fibrogenessis in IPF.[5–7] Hence, the prevention of MMP activity can therapeutically benefit the patient of IPF. Here, we present a small open trial of IPF patients (diagnosed on HRCT basis) treated with doxycycline, a non specific MMPs inhibitor.

## MATERIALS AND METHODS

This is an OPD based open single arm prospective trial of use of doxycycline in IPF. The patients were selected from the OPD practice of a pulmonologist and evaluated for the diagnosis of IPF with clinical, radiological (chest X-ray posteroanterior view and HRCT chest) and whenever possible examination of broncho-alveolar lavage to rule out any infection. They were asked to take doxycycline 100mg, twice a day, only upon signing the written informed consent.

The patients of IPF with, clinically suspected exacerbation, very sick status with baseline arterial oxygen saturation (SaO_2_) < 90% at rest in room air, severe shortness of breath or need for oxygen therapy at home, difficulty to perform outpatient follow-ups, were excluded. The patients showing extensive honeycombing in HRCT chest or unwillingness to offer the consent to undergo the treatment with doxycycline or having history of intolerance with the drug in the past were also excluded. Thus, out of 88 patients of ILD seen during June 2005-July 2006, we diagnosed 22 cases as IPF of whom 10 patients had given consent for undergoing treatment solely with doxycycline. All the patients were given histamine 2 receptor blocker (ranitidine) concomitantly to reduce the chance of gastric intolerance. There was no defined protocol for evaluation and follow up; they were done as per the free will of the treating physician and the convenience of the patients who were only insisted for coming to follow up regularly. The initial pulse rate and arterial oxygen saturation were recorded once the patient sat quietly for at least three minutes and relaxed. Then he or she was asked to walk a fixed and measured distance of 60 feet inside the consultation office to reassess the pulse rate and arterial oxygen saturation with the help of a pulse oxymeter to note the maximum change in the pulse rate and the arterial oxygen saturation during an observation in 60 seconds following the walk. The progress of the disease were noted from subjective impression as ‘better’, ‘slightly improved’/‘stable’, or ‘worse’; better being reduction in symptoms as cough and dyspnoea and improvement in functional capacity, ‘slightly improved’ or ‘stable’ meaning no change or not a confident improvement and ‘worse’ signifying subjective worsening in symptomatology compared to the previous visit. Objective assessment was done with, a) the pulse rate and the arterial oxygen saturation at rest and the changes after 60 feet of walking; b) radiological changes in chest x-ray (PA view); c) change in forced vital capacity (FVC) over time; and d) weight gain or loss. The radiological change in a patient was decided from the pooled opinions of the treating physician as well one radiologist and one different pulmonologist who were kept blind about the identity and the treatment of the patient. The observations have been done systematically according to a given protocol with the provision of recording the impression zone wise from the Chest x-rays and the change was noted considering the initial findings as 100%. The mean of the changes of the observations in terms of (%) percentage of improvement was taken for analysis. The patients were continued on doxycycline once they maintained a stable state or showed improvement as per office assessment. For any deterioration or no improvement or so called ‘stability’ or ‘slight improvement’ in more than two consecutive visits without any apparent infection or obvious cause, they were shifted to the standard protocol of IPF therapy as prednosolone (½ − 1mg/kg/day) ± azathiaprine (2mg/kg/day). The patients were requested to visit initially a little frequently (once in a fortnight or so) and then as and when necessary (preferably once in 6 to 8 weeks) without any defined protocol of follow up visits. They were also offered and shifted to conventional therapy as and when they desired so. The toxicities of doxycycline were looked for in all the cases especially for gastrointestinal intolerance (nausea, vomiting, dyspepsia, anorexia), skin rash or photosensitivity, mucosal candidiasis, and anaemia and thrombocytopenia. Enquiry has been made over telephone for all the subjects before analysis of the data but the facts in the last follow up visit were only incorporated for the statistical analysis. The statistical calculation has been done with the data available at the initial and the final (the data of the last visit considered) visit applying Student's ‘*t*’-test.

## RESULTS

A total of 10 patients (male: 8, female: 2), with a mean age of 70.10 ± 8.90 years, were recruited between June 2005 - July 2006. They were followed up as per their convenience. Three patients were shifted to standard treatment following a short period on doxycycline alone (when they opted to try the drug initially and later opted for standard therapy following a mean of 30 days without any improvement). Seven patients continued therapy with improvement. They were followed up for a mean period of 531.43 ± 328.88 days before the final analysis. Out of the seven patients, three continued it with irregular follow up, one continued without follow up and three stopped the drug and lost follow up. The average duration of therapy has been 837.66 days (i.e., over two years and four months) for the three patients who continued the therapy [Table 1]. The [Table 1] shows the details of the patients taking the drug and the [Table 2] elaborates the results of the intervention in terms of change in the different variables considered. There have been overall improvement in all the parameters considered but it has been found statistically significant only for radiological changes.

**Table 1:** Details of the patients incorporated in the case series with their co-morbidities, duration of treatment and follow up

Name	Age/Sex	Comorbidity	Duration of doxycycline (before calculation)	Status of doxycycline
AS	73/m	Hypertension	796 days	continued
		Obesity		
Am	70/F	Obesity	900 days	continued
		GERD		
NB	75/m	COAD	378 days	Stopped*
		Hypertension		
		Hypothyroid		
		Dyslipidemia.		
RNB	72/m	None	837 days	continued
RRC	84/F	Dm	545 days	Lost follow up #.
		OSA		(continued till Jan'08)
		IHD		
SM	73/m	Hypertension	73 days	Stopped**, shifted to standard therapy. (Died on Oct'07)
		Dm		
USS	60/m	Dm	191 days	Lost follow up***, expired

Mean Age: 72.43±7 .09 years. Mean duration of therapy as per the last follow up visit: 531.43±328.88 days COAD—chronic obstructive airway disease, GERD—gastro esophageal reflux disease, DM—diabetes mellitus, OSA—obstructive sleep apnoea, and IHD—ischemic heart disease

# RRC: Dropped follow up for logistic reasons following December 2006, but she continued the drug till January 2008 without having any deterioration. She had one possible lower respiratory tract infection for which she was treated at home by her family physician. She is still active and able to take care of her day to day activities without help.

* NB: lost follow up and stopped doxycycline following visiting a tertiary care center. He appears stable and not significantly deteriorated.

** SM: lost follow up after the second follow up visit; went to a tertiary center on request of friends where doxycycline was stopped and conventional therapy was started. He showed progressive deterioration and has passed away in October 2007.

*** USS: Stopped follow up after November 2006. He went to another city and probably continued treatment there but it is not known whether he had continued doxycycline or not. He died probably from lower respiratory tract infection with hemoptysis and sepsis in mid 2007.

**Table 2:** The effect of doxycycline therapy on different variables

Variables	When doxycycline was introduced			Last visit while on doxycycline			*t*-test	*P* value
				
	Range	Mean	Median	Range	Mean	Median		
Resting pulse rate (min)	60-94	79.14 ± 11 .75 (7)	78	61-100	75.43 ± 12 .49 (7)	74	0.57	>0.05
Pulse rate after exercise (min)	76-107	92 ± 11 .53 (7)	94	70-122	86.57 ± 16 .89 (7)	83	0.70	>0.05
Resting arterial oxygen saturation (%)	94-99	96.86 ± 1 .46 (7)	97	95-99	97.29 ± 1 .60 (7)	98	0.53	>0.05
Arterial oxygen saturation after exercise (%)	88-99	94.14 ± 3 .44 (7)	95	86-98	93.86 ± 3 .93 (7)	95	0.14	>0.05
Forced volume capacity (l)	0.96-2.89	1.96 ± 0 .97 (3)	2.04	1.03-3.06	2.06 ± 1 .02 (3)	2.1	0.12	>0.05
Weight (kg)	54-86	69.21 ± 12 .19 (7)	73	57-84	70.14 ± 10 .45 (7)	72	0.15	>0.05
Chest X-ray (PA view) involvement (taking 1^st^ visit X-ray as 100% involvement)		100 ± 0 (6)	100		73.06 ± 13 .06 (6)	70.83	5.05	<0.01*
	-	-	-		-	-	-	-

* statistically significant. There is improvement in all the parameters but it is significant only for the radiological changes (*P* < 0.01)

## DISCUSSION

IPF is a difficult to treat disease. The treatment options are practically restricted to steroid and an immunosuppressive drug alone or in combination. Hence, a lot of research has been focused to new drugs (γ-interferon, Bosantan, pirfenidone, antioxydants) that have raised more hope than success.[8–13] We have observed earlier a significant improvement in different parameters in a case of IPF with prolonged doxycycline therapy.[14] This stimulated us to further observe the effect of the drug in other cases of IPF.

The study shows that there has been an impressive improvement in chest x-ray (PA) and improvement, though not significant, in all the other parameters observed at the end of over 17 (531.43 ± 328.88 days) months of therapy with the drug.

The study has a lot of weaknesses. It is an open single arm prospective trial without a proper design, adequate evaluation criteria, a clearly defined protocol and with very small recruitment etc. It cannot match a properly conveyed double blind study or even a open prospective trial with all the parameters as DLCO, repeat HRCT, lung biopsy and others. To mention, especially, the functional status and the quality of life of the patients were not categorized objectively by tests such as 6 minutes walk test, Saint George's Respiratory Questionnaire (SGRQ) and alike. Incorporation of these functional parameters could have been better in terms of objective assessment of the effect of the drug. Here, the functional improvement was noted with the patients' impression in the usual practicing protocol of the physician concerned without any compulsion for detailed evaluation. Infact, some of the parameters mentioned were not uniformly measured in all the visits and, though sought for, there was no compulsion on the patients' compliance for evaluation and follow up.

Factually, beyond HRCT, most of the patients had definite financial and logistic problems to afford further evaluation. Although the HRCT chest is a reasonable diagnostic tool for IPF, a lung biopsy would have been better. The measurement of pulse rate and arterial oxygen saturation at rest and following walking a defined distance (60 feet) was observed uniformly as a substitute for established functional evaluation (6minute walk test). However, the incorporation of the measurement of respiratory rate was not considered for the lack of an objective measuring device although, to our opinion, the change in respiratory rate was the most marked clinical improvement. Though not validated for the purpose, the measurement of exercise induced desaturation appears a physiologically sound action. None of the patients who continued the therapy had severe disease clinico-radiologically.

Thus, despite limitations, the improvement observed in pulse rate and other parameters before and after a defined exercise is certainly an indicator of improvement of the disease. The small number of patients in the series is perhaps the most important deterrent to assess the significance of the improvement. We tried to reduce the personal bias of the treating physician regarding the radiological assessment by incorporating the opinion of two other observers who were blind about the patients' identity or therapy. The observation that the radiological changes showing higher significance also needs further validation and comparison to the standard form of therapy.

It is important to note that out of seven, four patients lost follow up and stopped medicine. Two out of them (SM and USS in Table 1) had the history of drug intake for 73 days and 191 days. The other two patients (NB and RRC, see Table 1) continued the drug for a longer duration (378 and over 900 days, respectively). Incidentally, the two patients with shorter duration of therapy with doxycycline expired, while the other two with relatively longer duration of therapy are still living and are reportedly functionally active. On the other hand, all the three patients continuing doxycycline (for over 837.6 days on an average) and follow up are thriving well without any deterioration (rather improvement) of the functional status according to our mode of measurements. Although it is not possible to comment regarding the effect of doxycycline on survival from this small observation, but this difference in mortality appears noteworthy in our opinion. Moreover, considering the life expectancy of the patients of IPF, the fact of having improvement (although not statistically significant in all the parameters incorporated) in patients with a fairly prolonged mean follow up of 531.43 (± 328.88) days and actual average follow up of 837 days (for those continuing the medicine), it is prudent to comment that doxycycline is probably effective and may render survival benefit to the sufferers of IPF on a long-term basis of the therapy. In our small series the drug also appears safe; no patient had any worth mentioning adverse event with doxycycline. It is possible that the co-administration of ranitidine could have prevented the gastrointestinal side effects and that, in an observation without a stringent protocol, some minor side effects could have escaped detection. Finally, keeping in mind the co-morbidities of IPF [Table 1] and considering the age of the patients, it should be highly appreciated once a treatment appears possible without worsening the co-morbid states and averting the toxicities of steroid and/or immuno-suppressants which are being used for long-term as the conventional and recommended therapy of IPF.

The prevailing pathophysiological concept of IPF has been recently shifting from inflammation induced fibrosis[15] to a disease of fibroblast proliferation and dysregulated fibrosis.[16] Abnormal lung remodeling ensues from accumulation of extra cellular matrix (ECM) following epithelial injury.[16] There is dysregulated activity of MMPs, a group of enzyme that can be related to release of fibroblast growth factor which is important for continuous stimulation of fibrogenesis in IPF.[17,18] An imbalance in matrix metalloproteinases and tissue inhibitors of matrix metalloproteinases (TIMPs),[17,18] delayed or absent reepithelialization[19,20] and increased vascularity[20,21] are noted in IPF. The fibrogenesis may evolve from enhanced degradation of basement-membrane matrix and reduced production of TIMPs. Hence, prevention of the MMP activity could be therapeutically beneficial for the patients of idiopathic lung fibrosis.

MMPs inhibition and resetting the MMP-TIMP relations following epithelial injury and inhibition of MMPs is one of the recognized targets of future therapy of IPF. Since doxycycline has been there in the market over 30 years, and its known property of MMPs inhibition being approved by the FDA, USA for periodontal disease.[22] We offered prolonged therapy with doxycycline for our IPF patients once they had subjective and objective improvement after a trial of 3-6 weeks. Prolonged therapy with doxycycline has been recorded well tolerated for over months in several conditions. Doxycycline can be used as a substitute for penicillin allergic patients.[23] In Q fever, endocarditis doxycycline was given for a mean duration of 55 months (median 60 months) in combination with ofloxacin, and for a mean duration of 31 months (median, 26 months) with hydroxychloroquin, where the patients were declared cured.[24] Similar experience of Q fever endocarditis has been described in a patient with biological prosthetic aortic valve and aortic homograft; the patient was successfully treated with doxycycline and chloroquin for two years.[25] Doxycycline is licensed for up to two years or more in the treatment of acne in the same dose as is used for malaria prevention. The UK Advisory Committee for Malaria Prevention (ACMP) has concluded that there is no evidence of harm in long-term use of doxycycline and it may be taken safely for periods of at least up to two years.[26] There has been a case report of successful treatment of apparently refractory pulmonary capillary endotheliosis with doxycycline probably exploiting the same mechanism of action.[27] Our patients who showed improvement to long-term doxycycline (more than 12 months) had no problem of tolerating the drug.[14]

The results of using doxycycline in our patients appear encouraging with respect to the effectiveness and lack of significant toxicities. Despite the lack of complex and standard objective evaluations, the improvement in simple but scientifically sound observations, to our mind, is important and worthwhile to share. We feel that this widely used and relatively safe drug can be an effective adjunct in the treatment protocol of IPF in future. Also, doxycycline merits serious evaluation through systematically organized studies in order to evaluate its role in the management of IPF.
